# Different Setting Conditions Affect Surface Characteristics and Microhardness of Calcium Silicate-Based Sealers

**DOI:** 10.1155/2018/7136345

**Published:** 2018-01-16

**Authors:** Dong-Kyu Yang, Sunil Kim, Jeong-Won Park, Euiseong Kim, Su-Jung Shin

**Affiliations:** ^1^Department of Conservative Dentistry, College of Dentistry, Gangnam Severance Hospital, Yonsei University, 211 Eonju-ro, Gangnam-gu, Seoul 135-720, Republic of Korea; ^2^Department of Conservative Dentistry and Oral Science Research Center, College of Dentistry, Yonsei University, 50-1 Yonsei-ro, Seodaemun-gu, Seoul 120-752, Republic of Korea; ^3^Department of Conservative Dentistry and Oral Science Research Center, College of Dentistry, Gangnam Severance Hospital, Yonsei University, 211 Eonju-ro, Gangnam-gu, Seoul 135-720, Republic of Korea

## Abstract

**Objective:**

To investigate the effect of different setting conditions on surface microhardness and setting properties of calcium silicate-based sealers.

**Methods:**

Three sealers, EndoSequence Bioceramic (BC; Brasseler USA, Savannah, GA, USA), Endoseal MTA (ES; Maruchi, Wonju, Korea), and Well-Root ST (WR; Vericom, Chuncheon, Korea), were compared. Specimens were exposed to either butyric acid (pH 5.4) or phosphate-buffered saline (PBS [pH 7.4]) for 48 h and stored at 100% humidity for 12 days. The control specimens were stored at 100% humidity for 14 days. Surface microhardness was measured, topographic changes were observed, and phase analysis was performed using X-ray diffraction. Microhardness according to storage conditions was compared using one-way analysis of variance and Tukey's multiple comparison tests (*P* < .05).

**Results:**

The BC and ES sealers exhibited decreased microhardness when stored in acid or PBS compared with control (*P* < .05). In the WR group, acid exposure lowered microhardness of the specimens compared with control (*P* < .05). Scanning electron microscopy revealed different topographies in specimens from all tested sealers exposed to acid or PBS.

**Conclusion:**

The surface microhardness of calcium silicate-based sealers was reduced by exposure to either acid or PBS. Acid solutions, however, had a more detrimental effect than PBS.

## 1. Introduction

Mineral trioxide aggregate (MTA; ProRoot, Dentsply, Tulsa, OK, USA) is a calcium silicate cement and has been reported to have long-term clinical success rates [1] due to good sealing ability, biocompatibility, and osteoconductivity [2–4]. Based on the success of this product, interest in using calcium silicate-based materials as sealers is increasing for root canal treatment, and various calcium silicate-based sealers have recently been introduced.

Among the sealers released, premixed and injectable type sealers are simple to use. EndoSequence Bioceramic (BC; Brasseler USA, Savannah, GA, USA) is one of these sealers that set using the moisture remaining after the canal is dried with a paper-point. It consists of calcium silicates, monobasic calcium phosphate, zirconium oxide, tantalum oxide, and thickening agents. It has been reported that BC sealers have high biocompatibility [5], a marginal adaptation similar to MTA [6], strong antibacterial properties [7, 8], and the ability to enhance osteoblastic differentiation of periodontal ligament cells [9] and induce dentin remineralization [10]. Endoseal MTA (ES; Maruchi, Wonju, Korea) is another calcium silicate-based sealer and was previously reported to induce dentinal tubule biomineralization [11]; it has suitable biological and physical properties [12], satisfactory cytocompatibility [13], and good sealing ability [14]. This sealer is a premixed type and absorbs moisture from the air. It is composed of calcium silicates, calcium aluminates, calcium aluminoferrite, calcium sulfates, radiopacifier, and thickening agent. Well-Root ST (WR; Vericom, Chuncheon, Korea) is a newly introduced calcium silicate cement and is composed of calcium aluminosilicate compound, zirconium oxide, filler, and thickening agent. To date, however, there have been no published studies regarding this particular material. According to its manufacturer, it requires the presence of water to set and harden.

There have been studies investigating microhardness as well as properties such as the biocompatibility of calcium silicate-based sealers. The microhardness of MTA and MTA-like materials was reduced by exposure to butyric acid, which suggests that these materials were not fully set [15]. Furthermore, Loushine et al. [16] reported that the microhardness of the EndoSequence BC sealer was reduced by mixing with water. During canal obturation, sealers are likely to contact fluid around the apex. Because calcium silicate-based sealers have compositions similar to MTA, the setting of sealers could also be affected by the adjacent environment. The adjacent tissue near the apex may have normal or acidic pH due to infection and inflammation [17]. The effect of setting environment on the properties of calcium silicate-based sealers has not been investigated. Considering this background information, the aim of this study was to measure and compare the microhardness of EndoSequence BC, Endoseal MTA, and Well-Root ST sealers in acidic, phosphate-buffered saline (PBS), and 100% humidity environments. Additionally, the surface of the specimens was examined.

## 2. Material and Methods

### 2.1. Sample Preparation

EndoSequence Bioceramic (BC), Endoseal MTA (ES), and Well-Root ST (WR) sealers were used and compared in this study. A total of 90 polyethylene molds, 4 mm in diameter and 2 mm in length, were prepared. One side of the molds was covered using a matrix band and sticky wax to enable the sealers to set without leakage. The sealers were slowly injected into the molds (30 specimens for each material). The first group from each material was stored at 37°C and 100% relative humidity for 2 weeks (14 days) and used as control. In the two other groups, samples were stored at 37°C and 100% humidity for 2 days in two different storage conditions, in which the samples were immersed with 1 mmol/L butyric acid (pH 5.4) or PBS (pH 7.4) and then stored under the same conditions as the first group for 12 days. The total number of specimens in each group was 10. After each storage period, specimens were separated from the molds using a disposable No. 15 scalpel blade by cutting the walls of the molds vertically. The samples then underwent grinding using #1200-grit silicon carbide sandpaper (CC-1200w, Daesung, Seoul, Korea).

### 2.2. Surface Microhardness Measurement

A Vickers microhardness tester (HMV-SHIMADZU, Kyoto, Japan) was used to measure the microhardness of specimens. The square-based and pyramid-shaped diamond indenter was used with a full lead of 98.07 mN, 98.07 mN, and 980.7 mN for the BC, ES, and WR sealers, respectively. The indentation time was 5 s at room temperature. The Vickers hardness number was calculated by the testing machine using the following formula:

(1) HV=2Fsin⁡136°/2d2,HV=1.8542Fd2,

where *F* refers to the load in kilogram-force, *d* represents the mean of the two diagonals in mm, and HV indicates the Vickers microhardness value. Values of microhardness were measured three times and the average was calculated.

### 2.3. Surface Examination

#### 2.3.1. Scanning Electron Microscopy (SEM)

The surface (in contact with solution) microstructure of the specimens was analyzed using SEM (S-4700, FESEM, Hitachi, Tokyo, Japan). Specimens were coated and voltage was set to 15.0 kV, the signal type was secondary electrons, the working distance was 12 mm, and the scan speed was 16 frames per 20 s. Images were acquired at 500x, 3500x, and 15,000x magnification.

### 2.4. X-Ray Diffraction (XRD) Analyses

The crystalline structure of calcium silicate-based sealers stored under three different conditions was analyzed using XRD.* The samples for XRD were prepared in the manner described above and they was dried before the specimen was scanned*. An X-ray diffractometer (Ultima IV, Rigaku, Tokyo, Japan) was operated at 40 kV and 30 mA with Cu-K*α* radiation. The scan size was 0.02° and scan speed was 2°/min. Peak matching was achieved using standard data in the powder diffraction files (PDF) from the International Center for Diffraction Data (ICDD). The phase fraction was analyzed using the whole-pattern fitting function included in Jade version 9 software (MDI, Livermore, CA, USA).

### 2.5. Statistical Analysis

One-way ANOVA and Tukey's multiple comparison tests were conducted to determine statistically significant differences in microhardness according to storage condition for the same material;* P* < .05 was considered to be statistically significant.

## 3. Results

### 3.1. Surface Microhardness Measurement

**Figure 1:**
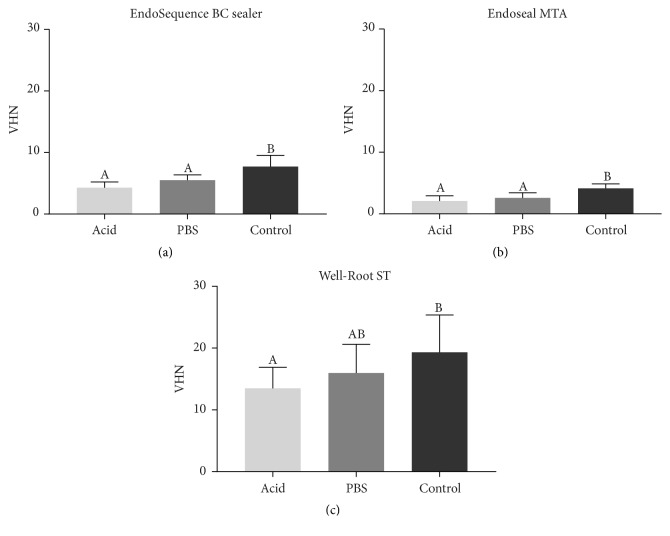
Mean surface microhardness of EndoSequence Bioceramic (BC; Brasseler USA, Savannah, GA, USA), Endoseal MTA (ES; Maruchi, Wonju, Korea), and Well-Root ST (Vericom, Chuncheon, Korea) under different setting conditions. The *y*-axis indicates the value of microhardness (VHN). One-way analysis of variance (ANOVA) and Tukey's multiple comparison tests were performed to compare each group with one another. Data are presented as means and standard deviations of each group and columns containing the same letter or letters are not statistically significant (*P* > .05).

Figure 1 summarizes the data from the surface microhardness measurements. In all tested materials, statistically significant differences in microhardness were revealed among the groups according to different storage environments (*P* < .05). Tukey's multiple comparison test demonstrated that the acid and PBS groups exhibited significantly lower mean microhardness compared with control in the BC and ES sealers (*P* < .05), whereas the WR sealer showed a significant difference between the acid and control groups (*P* < .05).

### 3.2. SEM

**Figure 2:**
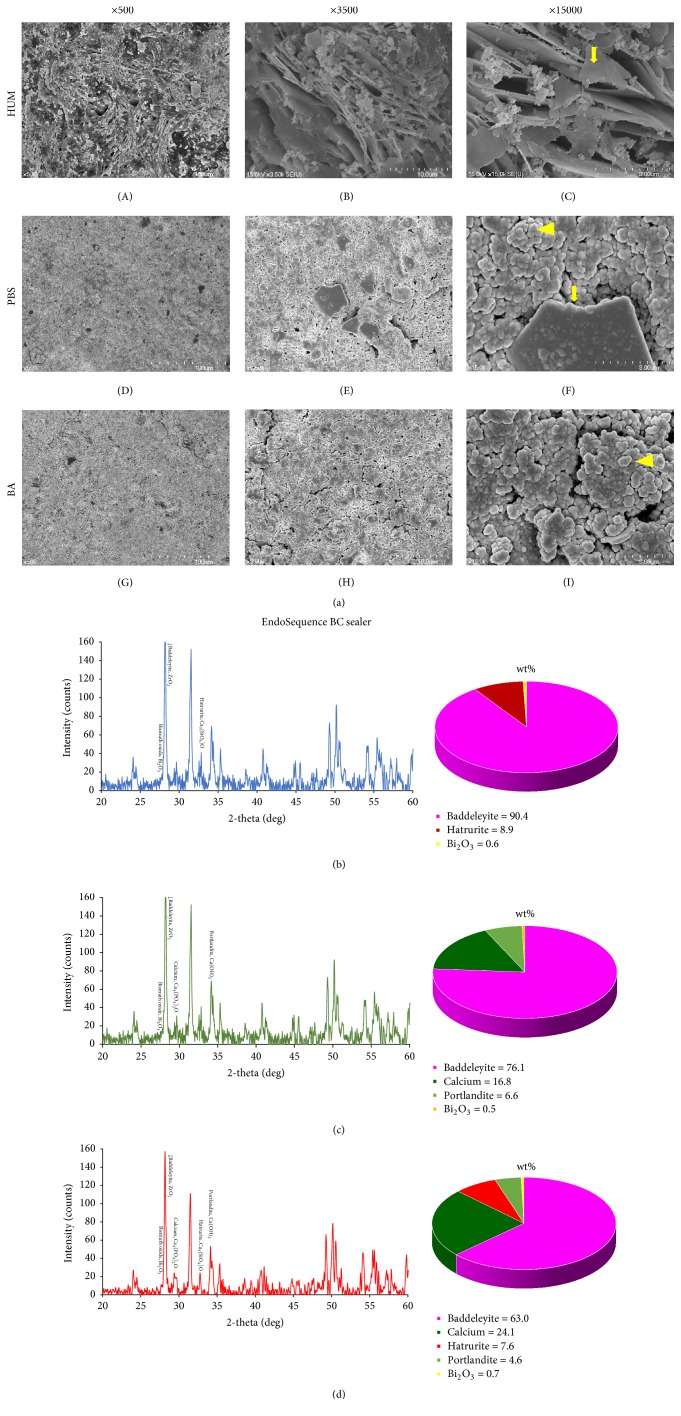
(a) Scanning electron microscopy images of EndoSequence Bioceramic (BC; Brasseler USA, Savannah, GA, USA) sealer in different setting conditions. (A), (B), and (C) Layered planar-like crystalline structures* (arrow)* were mostly found in the BC control group. (D), (E), and (F) High proportion of amorphous structures* (arrowhead)* with seldom crystallized* (arrow)* structures were found. (G), (H), and (I) The fewest number of crystals are apparent. Surface XRD analysis and phase fraction of the EndoSequence BC Sealer stored for 14 days in 100% humidity only (b), initially exposed to phosphate-buffered saline (PBS) for 2 days (c) and acidic solution for 2 days (d).

Different surface microstructure was observed in all tested materials according to setting conditions. The control in BC exhibited the most planar-like crystals (Figure 2(a)(C)), whereas more amorphous globular particles were observed in the PBS (Figure 2(a)(F)) and acid groups (Figure 2(a)(I)). More planar groups were found in the PBS specimen compared with the acid sample.

**Figure 3:**
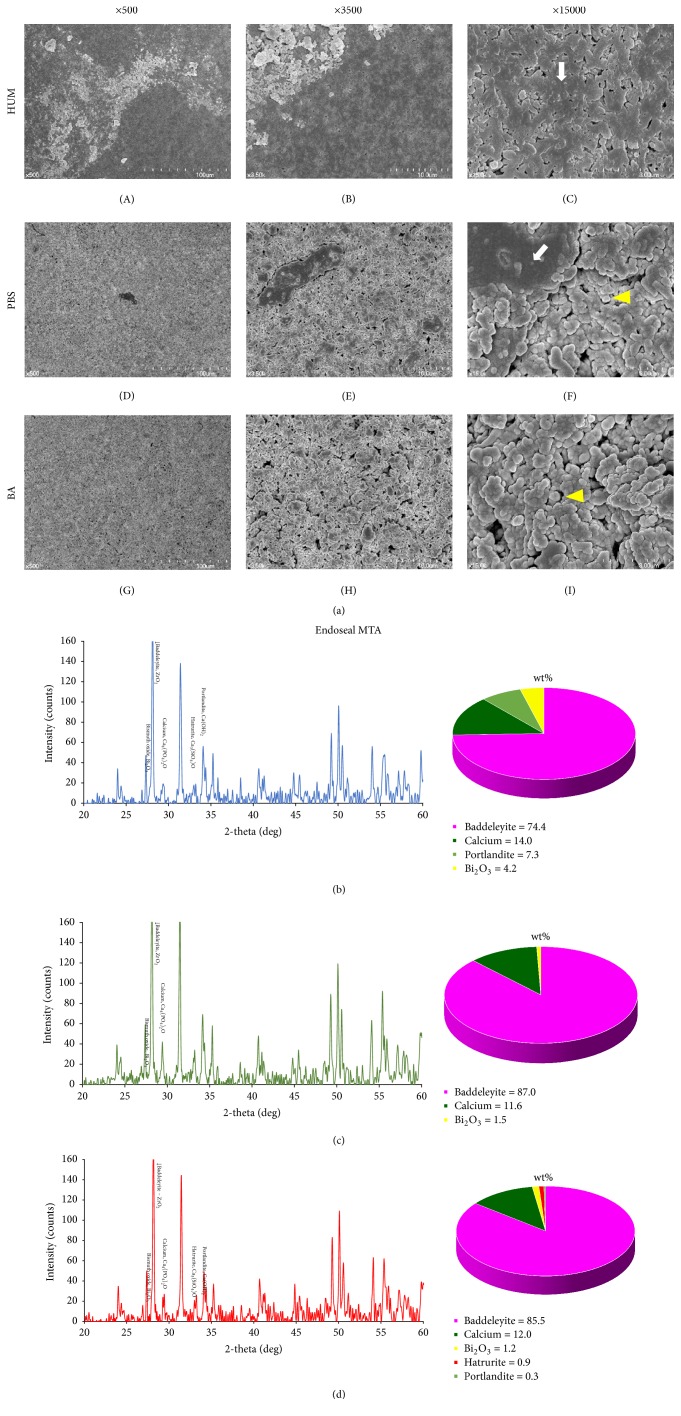
(a) Scanning electron microscopy images of Endoseal mineral trioxide aggregate (MTA, Maruchi, Wonju, Korea) under different setting conditions. (A), (B), and (C) All structures appear to be tightly connected with one another. (D), (E), and (F) A few clusters of globular particles* (arrow)*. (G), (H), and (I) No globular aggregate particles are apparent. Surface XRD analysis and phase fraction of Endoseal MTA stored for 14 days in 100% humidity only (b), initially exposed to phosphate-buffered saline (PBS) for 2 days (c) and acidic solution for 2 days (d).

In the ES group, no specimens exhibited crystallized structures (Figure 3(a)). However, in the control, clusters of globular particles formed on the surface of the specimen (Figure 3(a)(C)). The PBS group exhibited a small proportion of integration of the particles, similar to the BC sealer (Figure 3(a)(F)). The highest number of amorphous structures was observed in the acid group (Figure 3(a)(I)).

**Figure 4:**
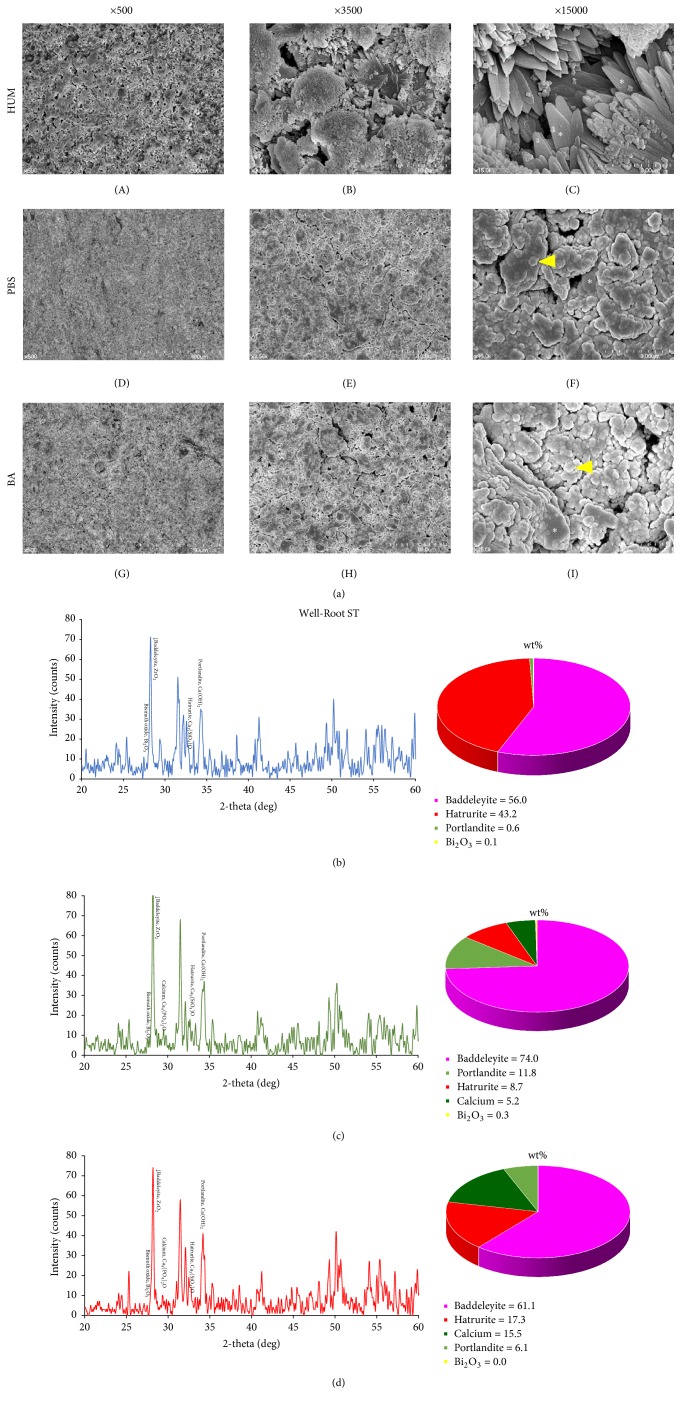
(a) Scanning electron microscopy images of Well-Root ST (Vericom, Chuncheon, Korea) under different setting conditions appear to be their primary particles and the degree of fusion differs depending on the condition. (A), (B), and (C) Large number of petal-like crystallized structures* (asterisk)*. (D), (E), (F), (G), (H), and (I) Clusters of globular particles* (arrowhead)* with petal-like crystals* (asterisk)*. Surface XRD analysis and phase fraction of Well-Root ST stored for 14 days in 100% humidity only (b), initially exposed to phosphate-buffered saline (PBS) for 2 days (c) and acidic solution for 2 days (d).

Significantly more crystalline structures were observed in all WR specimens compared with the other materials under the same conditions (Figure 4(a)). WR control samples exhibited a large number of petal-like crystallized structures (Figure 4(a)(C)), whereas the other WR groups exhibited similar surface characteristics (Figure 4(a)(F)–(I)).

### 3.3. XRD Analyses

XRD of the sealers under different conditions is shown in Figures 2(b)–2(d), 3(b)–3(d), and 4(b)–4(d). Zirconium oxide (ZrO_2_, PDF#01-080-0966) exhibited the strongest peak among all groups (Figures 2(b)–2(d), 3(b)–3(d), and 4(b)–4(d)). In the BC sealer groups, only the specimens immersed in acid and PBS exhibited tetracalcium diphosphate monoxide (Ca_4_[PO_4_]_2_O, PDF#01-070-1379) and calcium hydroxide (Ca[OH]_2_, PDF#01-080-0966) (Figure 2). Tricalcium silicate (Ca_3_[SiO_4_]O, PDF#01-073-2077) was evident only in the PBS and control groups of the BC sealer (Figures 2(c) and 2(d)).

Calcium was observed in all ES specimens (Figures 3(b)–3(d)). Substantially less zirconium oxide was found when the material was not immersed in either solution. Calcium hydroxide was also lower in this group (Figures 3(b)–3(d)).

Tricalcium silicate showed one of dominant peaks in the control group of the WR sealer (Figure 4(b)). The percentage of calcium and calcium hydroxide in WR sealers exposed to acid and PBS was higher than that in BC sealers; tricalcium silicate was also found in the acid group (Figures 4(c) and 4(d)).

## 4. Discussion

Recently, a variety of calcium silicate based sealers have been introduced, and interest in the single-cone obturation technique using calcium silicate-based sealer has been increased due to improved biocompatibility [8, 16, 18] and dimensional stability [19] compared with conventional sealers. With this technique, the volume of the sealer is more likely to increase compared with other techniques; therefore, characteristics of the sealer may have a greater impact on clinical outcome (s). Therefore, it is important to identify the physical and chemical properties according to the setting conditions.

The sealers were exposed to two different conditions in an attempt to simulate clinical situations during canal filling. Butyric acid (pH 5.4) was used to mimic an inflammatory environment because inflammatory tissues typically exhibit low pH (approximately 5.5) [20] and butyric acid is a byproduct of anaerobic bacterial metabolism [21, 22]. Healthy blood is slightly alkaline (pH 7.4) [23], and PBS simulates tissue fluid containing phosphate [24]. Therefore, PBS at pH 7.4 was used to mimic healthy conditions. The specimens stored at 100% relative humidity represented the control group.

The manufacturers of calcium silicate-based sealers report that the setting time of the EndoSequence BC, Endoseal MTA, and Well-Root ST sealers is 4 h to 10 h, 12 h 31 min, and 25 min to approximately 2.5 h, respectively. However, because these sealers need hydration to set, the amount of fluid in the surrounding milieu affects the setting time. It is known that setting is slow in a dry canal [25]. Loushine et al. [16] reported that setting required at least 168 days in a 100% relative humidity chamber, compared with 2.7 h in a water bath [19] and 22.3 h in Hanks' Balanced Salt Solution [25]. Therefore, to allow sufficient time for hydration and setting in the present study, total storage time was 14 days and immersion time was set at 2 days.

Microhardness reflects the resistance of materials to deformation under specific load. In itself, this property does not have any clinical significance for a sealer material; it was used in this study as an indirect measurement of material setting. Although several fundamental properties, such as tensile strength [26], modulus of elasticity [26], and crystal structure stability [27], can affect microhardness, it provides information about the progression and quality of the hydration process and indicates the extent of the setting reaction [20]. Therefore, the Vickers hardness test was used to determine the effect of three different conditions on the setting of calcium silicate-based sealers.

In this study, calcium silicate-based sealers that came into contact with acid and PBS exhibited lower microhardness. These results are in agreement with a previous study investigating a calcium silicate cement (e.g., ProRoot MTA). Kim et al. [28] reported incomplete setting of MTA with exposure to FBS for 4 days. They recommend fast-setting MTA because contact with periapical fluids is unavoidable. Another study reported that MTA Plus (Avalon Biomed Inc., Bradenton, FL, USA) in direct contact with fluids resulted in decalcification of calcium silicate hydrate and microcracking and leaching of calcium hydroxide [29]. The authors speculated that the reason for this was that setting was hindered and prolonged by the presence of phosphate and glucose in the solution.

Our SEM results (Figures 2(a), 3(a), and 4(a)) demonstrated that the control groups of the BC and WR sealers had more crystal-like structures, and the control group of the ES sealer exhibited more aggregated structures than other groups. Crystals of MTA are known as calcium silicate hydrate or “Portlandite” (crystalline calcium hydroxide) [20]. This crystal structure appears to have affected microhardness by creating interlocking [20].

In previous studies, the microhardness of MTA was reduced significantly by exposure to acidic solutions due to its more porous and less crystalline microstructure [15, 30]. Our results were similar, while no statistically significant difference was found between PBS (pH 7.4) and butyric acid (pH 5.4) solutions. However, in SEM, the most visible structures were amorphous in the PBS and acid groups, whereas the acid groups exhibited less planar-like crystals and clusters than the PBS groups. Therefore, we suspect that the results could have been influenced by the location of the indentation, even though we calculated the average of three separate measurements.

XRD was performed to detect changes in the major constituents and compounds of calcium silicate-based sealers under different conditions. In MTA specimens, tricalcium silicate is involved in early strengthening, and dicalcium silicate contributes in the late phase of hydration [31]. However, based on the limited results of this study, we could not find similar XRD patterns according to setting environments. Additionally, there was no correlation between surface microhardness and XRD patterns. We interpreted these results to mean that the different conditions only affected the exposed surface of the specimens, given that XRD is able to assess the entire specimen, not just the surface. Further studies are needed to analyze surface structure only, which could be altered depending on setting/storage conditions.* When calcium silicate based cement was immersed in simulated body fluid, the surface of this cement was changed by calcium phosphate deposition from 24-hour samples and similar findings were demonstrated in both HBSS and PBS [32].* Additionally, XRD cannot visualize amorphous reaction products, which comprise a large part of what is likely produced. Detection of tricalcium silicate is likely unconsumed material that did not react. Another limitation of this study was that the surface of the samples was grinded in the test for microhardness and XRD. Although grinding was minimal, this procedure removed the true surface layer of the samples that made contact with the surrounding milieu under different conditions.* Previous studies [33, 34] showed calcium phosphate and/or hydroxyapatite formation from MTA and other calcium silicate based cement when the samples were immersed in Hank's balanced salt solution (HBSS) for 28 days. However, they were not detected in the present study and other studies [25, 29]. It was speculated that the different materials and/or short immersing period (2 days) could show different results.*

The present study was the first to investigate the setting properties of premixed/injectable calcium silicate-based sealers while simulating different conditions in the periapical area. The surface microhardness of the sealers was reduced by exposure to acid or PBS. The acid groups exhibited a tendency to exhibit lower surface microhardness.* Clinically after the calcium silicate based sealer is applied in the root canal system during canal obturation, its surface properties can be affected by the periapical tissue environment. Based on the results, acidic condition such as periapical inflammation can change sealer's physical and or chemical properties and may have a detrimental effect on long-term sealing ability. However, this hypothesis has not yet been proven and further studies are needed.*

## 5. Conclusions

Contact with various environments elicited different surface microhardness of the EndoSequence Bioceramic, Endoseal MTA, and Well-Root ST sealers. These materials exhibited lower microhardness when they were exposed to acid and PBS. Acidic environments are believed to further weaken the materials.
